# The Role of Social Responsibility and Ethics in Employees’ Wellbeing

**DOI:** 10.3390/ijerph19148838

**Published:** 2022-07-21

**Authors:** Claudiu George Bocean, Michael Marian Nicolescu, Marian Cazacu, Simona Dumitriu

**Affiliations:** 1 Department of Management, Marketing and Business Administration, Faculty of Economics and Business Administration, University of Craiova, 13 AI Cuza Street, 200585 Craiova, Romania; 2 Doctoral School, University of Craiova, 13 AI Cuza Street, 200585 Craiova, Romania; nicolescumichaelmarian@gmail.com (M.M.N.); cazacumariantbm@gmail.com (M.C.); simonadumitriu1969@gmail.com (S.D.)

**Keywords:** social responsibility, organizational ethics, wellbeing, employees, community, structural equation modeling

## Abstract

Social responsibility (SR) is a concept or practice by which organizations take into account the interest of society by taking responsibility for the impact of their activities on all stakeholders. The SR of organizations implies ethical behavior concerning all stakeholders and a company’s commitment to the sustainable economic development of society. Organizational ethics is a set of written and unwritten codes of principles and values that govern decisions and actions within an organization. Ethics has a rather internal perspective, while social responsibility has a rather external perspective. This study examines the impact of social responsibility and organizational ethics on employees’ wellbeing. To perform the empirical analysis, we conducted a survey among 423 employees from Romanian organizations. Using the structural equation modeling, we analyzed the relationships between social responsibility, organizational ethics, and employees’ wellbeing, emphasizing the positive impact of ethical and responsible behavior of the organization on the employees’ wellbeing. The organization’s employees play a dual role: firstly, they are all internal stakeholders, and secondly, they are constituents of an external stakeholder essential for the organization—the community. The results show a significant positive influence of social responsibility and organizational ethics on employees’ wellbeing as a result of a responsible and ethical behavior in relation to the organizational stakeholders.

## 1. Introduction

The modern organization is an entity with a substantial social impact due to its ability to mobilize productive resources and create new wealth. However, the organization’s legitimacy depends not only on success in creating wealth but also on its ability to meet the expectations of the various stakeholders that contribute to its existence and success.

Social responsibility (SR) concerns implementing ethical behavior and attitude in the organization, providing a perspective on core values and organizational culture to promote responsible behavior towards staff. Organizational ethics (OE) influence practices in the field of social responsibility. It is in the interest of every organization to develop and incorporate elements of both OE and SR into its agenda, as the challenges of an increasingly globalized economy with stringent sustainability requirements will require an integrated approach of OE and SR to support the sustainable development of organizations [1,2].

To be sustainable, organizations need to identify innovative ways to balance the social and environmental needs of internal and external stakeholders (employees, unions, community) with the economic (financial) needs of internal and external stakeholders (shareholders, employees, suppliers, customers, tax administrations) [3,4,5]. External SR extends to the community and society, including environmental concerns, while internal SR addresses the organization’s human resources [6]. In addition, internal SR focuses on strategies and practices to improve employee health and wellbeing (WB) [7], human rights [8], training and development [9,10], ensuring equal opportunities in business [11], and work–life balance [12].

Although most studies show a significant relationship between SR and OE practices, these relationships are neither universal nor consistent [13]. Therefore, investigating the different dimensions of SR practices concerning the dimensions of OE is necessary to integrate the two concepts and evaluate the combined effects on the employees’ WB and the community in which the organization operates.

Although the impact of SR and OE on economic, social, and environmental performance has long been analyzed, not many studies examine the effects of SR and OE on employees’ WB. Despite the awareness that employees are a key internal stakeholder whose motivation depends on the organizational success, being at the same time a constituent part of a critical external stakeholder (the community in which the organization operates), there are a few studies in the area.

The research gap that the paper aims to cover comes from the lack of work to study the combined effect of OE and SR on employees’ WB. Since organizational employees are an essential category of internal stakeholders, the organization must pay special attention to SR and OE; these significantly affect employees’ WB. This study’s objectives involve analyzing the direct relationships among employees’ perceptions of SR, OE, and WB, and the mediation effects between the variables considered. By studying these objectives, this study aims to understand better cause-and-effect relationships on how SR and OE can influence employee WB. The paper structure has six sections. The introduction and literature review approach the research topic from a theoretical point of view. Section 3 and Section 4 describe the research design and results. The last two sections provide discussions and conclusions of the research.

## 2. Literature Review

### 2.1. Social Responsibility 

SR is the moral responsibility of an organization toward the community in which it operates in particular and towards society in general [4,14]. SR is a concept that has received multiple definitions, and there are various classifications of its dimensions: the economic, legal, ethical, and philanthropic dimensions [15] and the economic, social, environmental, stakeholder, and volunteer dimensions [16]. Davis and Blomstrom argue that the substance of SR stems from the ethical “obligation” of the organization to assess the effects of its decisions and actions on the entire social system [17]. At the same time, [18] identifies the gap between the concept of SR and practice. Other authors [15,19,20] looked at SR in terms of organizational efforts to meet the needs of different categories of stakeholders. For example, McWilliams and Siegel [21] saw in SR an increase in the social interests of business organizations or a commitment to increase the reputation and improve the image by diminishing the community’s negative perception of the organization [22]. Matten and Moon considered SR to be a component of the organization’s strategic policy that illustrates its interest in social issues, not just the primary goal of profit maximization [13]. Aguinis considers that SR represents those actions and policies that meet stakeholders’ expectations to maximize results in three areas: economic, social, and environmental [6].

An issue increasingly addressed by an employer is employee involvement in SR actions [23]. Such employer behavior brings social benefits and plays an essential role in ensuring employees’ WB, directly affecting the satisfaction, commitment, and loyalty of current employees and leading to greater motivation, increased productivity, and a greater propensity to innovate [24,25]. In addition, when employees identify the organization’s commitment to socially responsible behavior, they tend to have more responsible attitudes that correlate with better performance due to improved relationships between employees and other stakeholders [26].

### 2.2. Relationships between Organizational Ethics and Social Responsibility

According to [27], philanthropic responsibilities stem from the philosophical, ethical tradition of concern for what is good for society and justify organizations to help improve the quality of life of different stakeholders and the community. Reich points out that SR is nothing more than intelligent management covered by the language of morality and ethics. Only organizations which aim to adhere to all universally accepted ethical standards can expect a positive attitude and support from society [26,28]. Moreover, solving the problems that affect the community and society leads to competitive advantages for the organization. Nord and Fuller saw corporate SR as a matter of higher-level strategy. They linked it to the conceptualization of organizational change, raising awareness of an alternative model that would complement the strategic vision and add an ethical dimension [23,24,29].

At the same time, managers have developed practices related to OE and SR within their organizations. There are many reasons why organizations implement these practices: reducing costs, mitigating risks, gaining legitimacy, gaining a competitive advantage, and creating new value [30]. In addition, researchers and managers have recommended aligning these practices within organizations [23,24,26,31,32,33,34]. Still, there is little empirical research exploring the impact of alignment or why it has not become a common practice within organizations. Based on these considerations, we formulated the following research hypothesis:

**Hypothesis** **1** **(H1).** *Employees’ perception of OE directly positively affects employees’ perception of SR.*

### 2.3. Employees’ WB

The community includes individuals in constant interaction in a particular space where they live and work [35]. In addition to the spatial dimension, a community may be determined by the common interest of its members [36]. Given that interactions between individuals within the community include several dimensions (psychological, cultural, spiritual, social, economic, and natural) [37], meeting all the needs of individuals related to these dimensions confers a WB status. Consequently, WB also includes the social, economic, environmental, cultural, and political dimensions [38].

The concepts of health and WB are often used together and sometimes even interchangeably. However, health refers to an individual’s physiological or psychological indicators [39], while WB is a more comprehensive concept that aim to describe the individual’s general condition in a social context [40,41]. Therefore, WB consists appropriately of non-contextual measures of life (e.g., life satisfaction, happiness), general considerations (e.g., job satisfaction), and more specific dimensions (e.g., salary satisfaction, good workplace).

WB includes the individual’s general satisfaction regarding privacy, social relationships, work environment, and reduced stress [42,43,44]. Therefore, employers’ concern for ensuring a better job for their employees and a WB status was considered a component of SR, which is part of ethical behavior.

The concept of WB has therefore been approached in the paradigm of the multidimensionality of human, social, and economic capital [45]; physical, psychological, social, and economic WB [46]; and social, environmental, economic, health, political, physical, and residential dimensions [47]. The economic dimension is manifested by providing sufficient income, job stability, and existing opportunities in the labor market [47,48]. The social dimension includes income and profession that offer a certain social status [37,49] and concepts such as security, community spirit, cohesion, trust, reciprocity, involvement, and informal interaction [5,37,47].

Employers want to improve employee wellbeing because lowering WB can lead to unhappiness, decreased productivity, and increased stress and anxiety, eventually leading to a high turnover [44,50,51,52]. Therefore, as a dimension of relationships and social status, employees’ WB can be considered an objective of SR concerning its human resources and work environment [53].

The WB concept integrates employees’ status at and outside the workplace: job satisfaction or dissatisfaction, reward, working relationships, working conditions, friendly work environment, promotion opportunities, care for the environment, and interest in the general health community. WB is a complex and multifaceted construct [54], balancing between objective indicators (life standards) and subjective measures (psychological, social, and spiritual aspects) [55].

Other authors have added to the social dimension the interaction between individuals in the family, at home, and in neighborhoods [56] or education [38]. The environmental dimension includes the perception of individuals about the place where they live, with a solid psychological load for individuals. McCrea et al. [47] suggested that environmental satisfaction, green areas, transport, air quality, energy quality, and sustainability are crucial indicators of WB [37,45,47,57,58,59].

### 2.4. Relationships between Employees’ WB, SR, and OE

SR is a social obligation of the organizations to decide and act responsibly following the objectives and values of society [60]. Currently, SR is perceived as a continuous commitment of organizations to behave ethically and contribute to the economic development of the community and society in which the organization operates by improving the quality of human WB, through involvement in the local community and society. SR is the basis of sustainability, competitiveness, and innovation and is a strategic advantage of any organization [61,62,63,64]

Due to the potential impact of organizations on WB employees and the community in which they operate, ethical behavior and SR programs are of great importance for overall WB [65]. In this context, Chowdhury et al. proposed an SR and OE reporting on stakeholder health and WB [66], based on the Global Reporting Initiative (GRI) sustainability reporting standards. Cheng et al. [67] suggest that if SR activities do not live up to employees’ expectations, they generate mistrust of organizations, leading to reduced commitment [34,67,68] and WB and increasing turnover rates [67]. Various authors [67,68,69,70,71] have studied the impact of employees’ perceptions regarding CSR and organizational ethics on outcome measures: employee satisfaction, turnover rates, and overall organization sustainability. Consequently, examining and monitoring employees’ perceptions regarding SR and OE is beneficial for the organization’s human resources management and strategic management to meet the expectations of all stakeholders, especially employees [67,68,69,72].

Based on the relationships between OE, SR, and WB described in the literature, we formulated the following research hypothesis:

**Hypothesis** **2** **(H2).** *Employees’ perception of SR and OE directly positively affects employees’ perception of WB.*

Internal stakeholder-oriented SR programs target WB employees by obtaining employee satisfaction based on meeting the expectations of their organizations [73]. Employees have an ethical expectation towards their organizations in terms of job stability, recognition and appreciation, fairness of rewards, opportunities for professional and personal development, freedom of association in trade unions, work–life balance, involvement in decisions, autonomy, participation in organizational decisions, and involvement of the organization in the community [74]. In addition, organizations will invest in ethical health and safety management practices that impact the company’s performance [75].

Occupational health and safety (OHS) promote human resource management, safety, occupational safety, physical and mental health, and in general, an essential part of the WB of human resources [76,77,78,79]. WB incorporates the employee’s physical, emotional, and mental wellbeing, exerting a significant positive impact on achieving objectives [74,77,80,81]. However, several authors [82,83] have highlighted the need to see the health and wellbeing of employees beyond the work environment by taking into account other ethical factors related to other areas of human resources: the process of training and development [9,10], ensuring equal opportunities in business [11], work–life balance [12], job stability, and existing options in the labor market [47,48]. 

Researching employees’ perceptions and attitudes towards SR, OE, and WB is important [34,84,85] because it can lead to seeking opportunities for better implementation of responsible and ethical social practices and initiatives. In addition, companies are increasingly recognizing the strategic importance of OE and SR in ensuring employees’ WB and the sustainability of their business [69,84,86], as well as employee satisfaction in implementing SR programs and ethical conduct.

Based on these considerations, we formulated the following research hypothesis:

**Hypothesis** **3** **(H3).** *Employees’ perception of OE has significant indirect positive effects on their perception of WB, mediating their perceptions of SR.*

**Figure 1:**
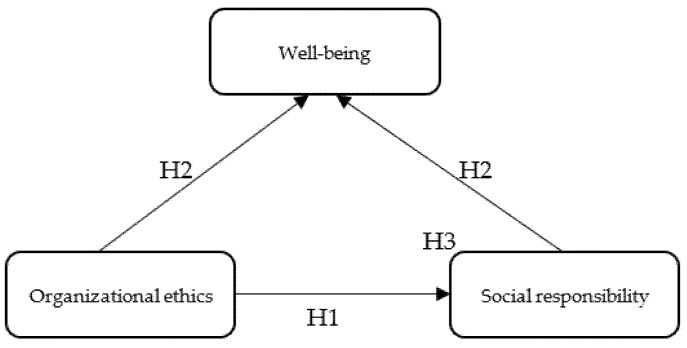
Conceptual model. Source: designed by authors.

Figure 1 shows the conceptual model of the research on the relations between SR, OE, and WB.

## 3. Methodology

### 3.1. Research Design

To study the impact of SR and OE on employees’ WB, we conducted quantitative research in a survey among employees of Romanian companies.

**Figure 2:**
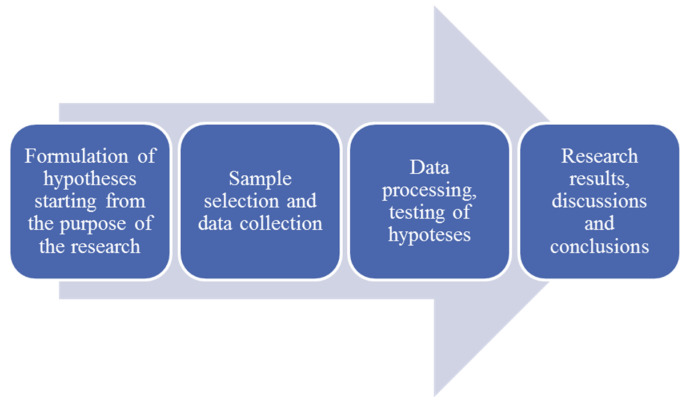
Research process. Source: own construction.

The data collected in a database were subjected to descriptive and inferential statistical analyses. To determine the intensity and meaning of the relationships between the research variables, we used structural equation modeling and artificial neural network analysis. Finally, the obtained results confirmed the hypotheses’ validity based on the literature. Figure 2 illustrates the research process.

### 3.2. Selected Sample

To perform the empirical analysis, we conducted a survey based on a questionnaire filled by 423 employees from Romanian organizations, small and medium enterprises, and large corporations between March 2022 and May 2022. The sampling method chosen was random stratified sampling. The target population of the research is the employees in Romanian private companies, comprising 4,500,000 individuals. The sample of 423 individuals was selected with a level of confidence of 95%, with a margin of error of 4.762%. Table 1 describes the descriptive statistics for the selected sample.

Employees were selected in the sample using the economic sector criterion: 9.9% in agriculture, 29.8% in industry, and 60.3% in services (including technology and communications). The sample structure according to the size of the companies from which the employees come is as follows: 40% of the employees come from small and medium companies, 40.2% come from large companies, and 19.8% come from multinational companies. Within the sample, 68.15% are male and 31.85% are female. Regarding the age, 9.95% are under 30 years old, 69.93% are between 31 and 55 years old, and 20.12% are over 55. In addition, 19.8% of respondents have received secondary education, and 80.2% have studied a higher degree. Over 60.3% of respondents have more than ten years of work experience, and over 60.2% have more than ten years of experience in the organization. Most respondents are subordinates, with only 19.81% being managers. Depending on the income category, over 43.32% of respondents have a net income above the average net salary in the economy.

### 3.3. Research Tools and Methods

The design of this study involved conducting a survey based on a questionnaire applied to employees of Romanian organizations. The questionnaire contains the socio-economic-demographic variables that characterize SR, OE, and WB. We evaluated the impact of SR and OE on WB empirically by using statistical methods for modeling structural equations (SEMs) in the partial least squares (PLS) variant using a procedure described by [87,88], similarly used by [89,90]. The initial literature review established measures for each construct and the reliability and validity of variables using various statistical tests (Cronbach’s Alpha, Composite Reliability, and Average Variance Extracted). We built items for SR and OE based on previous research. SR includes five dimensions describing the levels of responsibility: responsibilities to shareholders (increasing the organizational value); responsibilities to employees, unions, customers, and suppliers (societal welfare, organizational SR philosophy); responsibilities to central and local public authorities and the community (organizational citizenship); and responsibilities to society (societal contribution). The items concerning SR, which define the levels of responsibility, were defined based on [15,91,92,93]. OE includes five dimensions describing the ethical principles in the organization: transparency, fair competition, respect for the customer, employees’ wellness, and sustainability, as stated in other research [8,15,33,89]. The WB scale was established based on the TINYpulse questionnaire [94], using the eight dimensions for WB: general WB, emotional WB, environmental wellness, intellectual WB, occupational WB, physical health, and social WB. To measure the variables SR, OE, and WB, we used a five-level Likert scale (5—total agreement, 4—partial agreement, 3—agreement, 2—partial disagreement, 1—total disagreement).

The exogenous variables (the items of the questionnaire) which characterize SR, OE, and WB are presented in Table 2. 

The self-administered questionnaire results can be affected by common method bias (CMB) [95]. We tested all variables using Harman’s single-factor test using principal component analysis. The extracted variance was below 50% (45.329%), attesting to no significant common method bias effects [95].

## 4. Results

**Figure 3:**
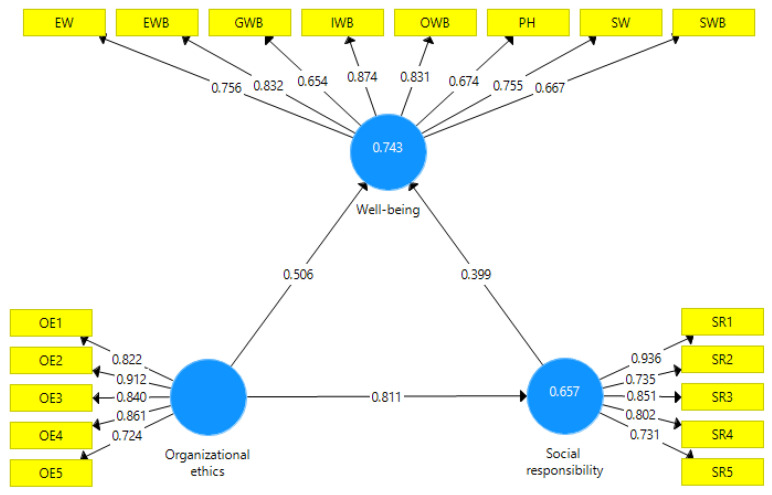
Preliminary model. Source: designed by authors using SmartPLS 3.0 (SmartPLS GmbH, Oststeinbek, Germany).

We used structural equation modeling (SEM) in the partial least square variant (SmartPLS 3.0 software: SmartPLS GmbH, Oststeinbek, Germany) to validate the three hypotheses. The model has three unobservable latent variables: SR, OE, and WB. Each of the three latent variables depends on a series of observable exogenous variables defined by the items in the questionnaire. Figure 3 shows the exogenous variables for each latent variable. 

**Figure 4:**
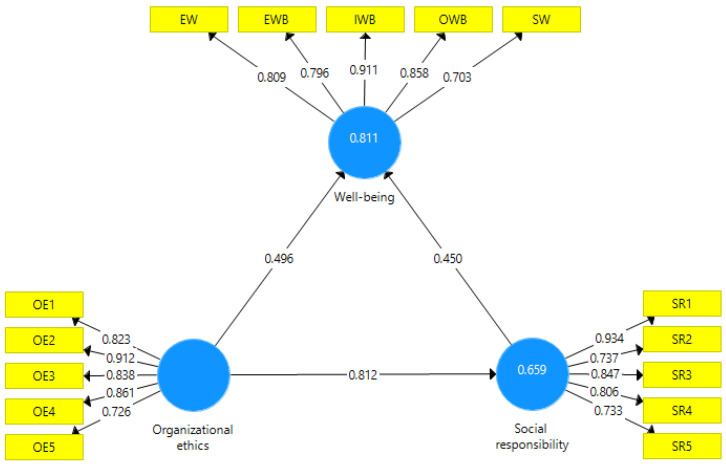
Model applied. Source: designed by authors using SmartPLS 3.0 (SmartPLS GmbH, Oststeinbek, Germany).

Following the methodology described by [88], we eliminated from the model those items that have an outer loading below 0.7, considering the lower influence of these items on the latent determinant variables. Figure 4 presents the resulting model.

The validity and reliability of the model were tested following the procedure described in [87,88]. All three indicators, namely Cronbach’s Alpha, Composite Reliability, and Average Variance Extracted), recorded good values (Table 3). In the model, SRMR recorded a value of 0.048, and NFI recorded a value of 0.934.

Finally, running a bootstrapping process, we determined the path coefficients and specific indirect effects in our model for assessing the role of SR and OE in ensuring employees’ WB (Table 4).

Analyzing the path coefficients and specific indirect effects in Table 4, we affirm that all three hypotheses are valid. The organizations’ ethical practices positively affect SR programs in employees’ perception. The two sustainability constructs (OE and SR) positively impact employees’ WB. In addition to the direct effect on WB, the organization’s ethical behavior has substantial indirect effects on WB, with an SR program based on ethical principles and values as a mediating variable.

## 5. Discussions

The relationship between SR, OE, and WB has not frequently been subject to an evaluation process in the literature on SR and OE. However, there is a recognition that this relationship can contribute to establishing sustainable jobs to ensure WB at the individual level and welfare at the societal level. In recent years, several researchers have conducted empirical studies to determine the impact of SR programs on work results from the perspective of stakeholders (including employees) [34,96,97,98,99]. Employees are key stakeholders who, once satisfied, can positively influence the implementation of SR programs [97]. Therefore, employees’ perceptions of SR shape the community’s view of organizations [96]. In addition, employees with a good level of WB can improve and stimulate SR programs and ethical behavior that promotes all stakeholders’ wellbeing, including employees [34].

Employers improve employee WB because low WB can produce unhappiness, lower productivity, and increased stress and anxiety, eventually leading to a high turnover rate [44,50,51,52,67]. Therefore, employees’ WB is an objective of SR programs concerning its human resources and work environment [53], ensuring employee commitment [68]. Researching the relationships between the variables of the researched model, SR and OE can contribute to increasing economic, social, and environmental performance and the health and wellbeing of employees, as we have demonstrated by confirming the validity of the H2 hypothesis.

The conceptual model in this study, which reveals the relationship between corporate SR and OE, also aims to help integrate and facilitate the implementation of SR activities and tools to ensure ethical conduct in organizations. Various authors have pointed out the need for a unified theory regarding SR and OE because there is much confusion and redundancy between the dimensions of the two concepts [27,30,33,89]. In our research, we tested the relationship established between SR and OE by confirming the validity of the H1 hypothesis. Combining these two areas can provide sustainability to organizations and ensure employees’ WB and that of the community they operate in [20].

Many studies have attempted to understand the impact of SR and ethical practices on employees’ satisfaction, a constituent of employees’ WB [34,84,85,100,101,102,103,104,105,106,107]. Researching employees’ perceptions and attitudes towards SR, OE, and WB is important [34,84,85] because it can lead to seeking opportunities for better implementation of responsible and ethical social practices and initiatives. In addition, employees’ satisfaction provides an insight into the emotional state of work experience and environment [108], directly contributing to organizational performance [73]. Although employees’ satisfaction is an essential component of employee WB, it is not just about satisfaction. There are several areas of SR that address job satisfaction aspects: job stability; employee status; fair pay; social benefits; occupational safety and health; work–life balance and employment opportunities; training and personal development; cordial labor relations; and a work environment characterized by communication, transparency and social dialogue, equal treatment, and equal opportunities [34,75,84,109]. Satisfaction is directly related to work, while WB also covers general aspects of general physical and mental health, relationships in the social environment, social status, care for the environment in which they live, and the individual’s connection to the community and society in general.

Programs in the SR area stimulate the improvement of health, the environment, and involvement in educational activities, acting as an essential mechanism for mediating between the organization’s ethical practices and improving employees’ and communities’ WB [110], as demonstrated by the confirmation of the validity of Hypothesis H3. Companies are increasingly recognizing the strategic importance of OE and SR in ensuring employees’ WB and the sustainability of their business [69,84,86], as well as employee satisfaction in implementing SR programs and ethical conduct. Organizations that promote health and safety management practices and ensure an adequate work environment [88] benefit from increased employee engagement, as the organization demonstrates an interest in employees’ WB. Rela et al. showed that other factors, such as community capacity and motivation, government policy, and other stakeholders’ contributions, influenced WB [5].

The results of our research are in line with the results of previous research showing that ethical issues can have a significant impact on physical health and spiritual wellbeing.

## 6. Conclusions

The research results indicate that the variables SR and OE have significant and positive influences on WB dimensions, consistent with previous studies showing a significant relationship between these constructs [4,111,112,113,114,115,116,117]. SR contributes to the satisfaction of employees’ interests related to WB dimensions (health, education, economy) and OE by inducing ethical behavior and attitudes that contribute to increasing WB. Research results confirm that SR programs and ethical behavior contribute to the employees’ wellbeing.

### 6.1. Practical Implications

Although OE activities and SR programs target both stakeholders, the present research focused on critical internal stakeholders (employees), given their dual nature. Employees are also constituents of an essential category of external stakeholders—the community. The research results confirmed the importance of SR and OE for improving employee wellbeing, SR being a mediating factor between OE and WB. These results support an essential mechanism by which OE activities and SR programs can increase WB, especially when the organization does not have sufficient resources to motivate employees and ensure job satisfaction. Employee satisfaction with job stability issues, guaranteeing a friendly work environment, caring for the environment in which they live, and organizational involvement in community social causes can all contribute to the overall WB of employees.

### 6.2. Theoretical Implications

Three issues can be highlighted as theoretical implications of this research. First, most studies have focused on external stakeholders [118,119,120,121], with few focusing on the positive effect of OE activities and SR programs on internal stakeholders. Second, while many types of research have addressed various facets of wellbeing (psychological, health, occupational wellbeing, etc.) [118,122,123,124], this study aimed at a holistic approach to the concept of WB. Although SR depends on the macroeconomic and organizational context, the main expectations for organizations are reducing poverty in the community and society in general, caring for the environment, improving public health, increasing employee WB, and an increasingly efficient educational process.

### 6.3. Limitations and Recommendations for Future Research

The analysis revealed a direct positive effect of SR and OE on employees’ WB. However, organizational ethics have a significant indirect positive impact on WB through SR programs that induce ethical conduct and the attitude of employees. These results should take into account various limitations of the research. First, the research only targets a category of stakeholders (employees) with a dual nature (internal and external) by their presence in the organization’s community. Secondly, the research was carried out only among the employees of some Romanian organizations, making it impossible to consider cultural differences between employees from different countries. Finally, the transversal approach to research provides more information through the results obtained, but does not offer a perspective on the evolutions of perceptions over time as a longitudinal approach.

Future research may address some of these limitations. In addition, future research may focus on studying the effects of moderating factors, such as communication, reputation, and organizational culture. Furthermore, there is a need for a deep investigation of the OE practices’ integration and alignment with SR programs to support a more synergistic impact on WB.

## Figures and Tables

**Table 1:** Descriptive statistics.

	Min	Max	Mean	Std. Deviation	Skewness	Kurtosis
Economic sector	1	4	2.81	0.981	−0.239	−1.048
Size	1	3	1.80	0.748	0.345	−1.146
Gender	1	2	1.30	0.459	0.875	−1.241
Age	1	5	2.70	1.099	0.606	−0.250
Education	1	5	3.30	1.101	−0.615	−0.237
Experience in work	1	5	2.30	1.345	0.669	−0.762
Experience in organization	1	5	2.91	1.136	0.187	−0.752
Position	1	2	1.20	0.399	1.517	0.301
Income category	1	5	2.91	1.512	−0.012	−1.443

Source: designed by authors using SPSS v.20 (SPSS Inc., Chicago, IL, USA).

**Table 2:** Exogenous variables.

Latent Variables	Exogenous Variables	
	Code	Description
WB	GWB	General WB
	EWB	Emotional WB
	EW	Environmental wellness
	IWB	Intellectual WB
	OWB	Occupational WB
	PH	Physical health
	SWB	Social WB
	SW	Spiritual wellness
OE	OE1	Transparency
	OE2	Fair competition
	OE3	Respect for the customer
	OE4	The organization treats employees well
	OE5	Sustainability
SR	RS1	Organizational citizenship
	RS2	Societal contribution
	RS3	Societal welfare
	RS4	Organizational SR philosophy
	RS5	Increasing the organizational value

Source: designed by authors based on [75,76,77,78].

**Table 3:** Validity and reliability.

	Cronbach’s Alpha	Composite Reliability	AVE
Organizational ethics	0.889	0.919	0.696
Social responsibility	0.875	0.907	0.664
Wellbeing	0.879	0.910	0.670

Source: designed by authors using SmartPLS 3.0 (SmartPLS GmbH, Oststeinbek, Germany).

**Table 4:** Path coefficients and specific indirect effects.

	Original Sample	Standard Deviation	T Statistics	*p* Values
Organizational ethics → Social responsibility (H1)	0.812	0.011	71.746	0.000
Organizational ethics → Wellbeing (H2)	0.496	0.033	15.031	0.000
Social responsibility → Wellbeing (H2)	0.450	0.033	13.462	0.000
Organizational ethics → Social responsibility → Wellbeing (H3)	0.365	0.023	15.637	0.000

Source: designed by authors using SmartPLS 3.0 (SmartPLS GmbH, Oststeinbek, Germany).

## Data Availability

Not applicable.
